# Considering evolved heat tolerance helps predict the immunological consequences of climate change

**DOI:** 10.1007/s00281-026-01082-z

**Published:** 2026-10-13

**Authors:** Jordan M. Lucore, Andrew J. Marshall

**Affiliations:** 1 Department of Anthropology, University of Michigan, Ann Arbor, MI USA; 2 Department of Ecology and Evolutionary Biology, University of Michigan, Ann Arbor, MI USA; 3 Program in the Environment, University of Michigan, Ann Arbor, MI USA; 4 School for Environment and Sustainability, University of Michigan, Ann Arbor, MI USA; 5 Program in Computing for the Arts and Sciences, University of Michigan, Ann Arbor, MI USA

**Keywords:** Climate change, Immunity, Immune system, Heat tolerance, Thermal limits, Evolutionary framework

## Abstract

Among the most damaging anticipated consequences of climate change are rising global temperatures that are expected to exceed the heat tolerance of many species worldwide. Species’ heat tolerance reflects adaptation to their historical environments, and rapid increases in ambient temperatures create a mismatch between evolved thermal tolerance and present-day heat exposures. This mismatch has severe implications because high temperatures can disrupt physiological processes across levels of biological organization. The immune system, specifically, may be particularly susceptible to dysregulation under climate warming because it is highly responsive to temperature. Although a rich body of empirical and clinical research has characterized the effects of heat on immunity, these findings are rarely interpreted in the context of evolved heat tolerance. Here, we provide a framework for predicting the immunological consequences of climate change by integrating results derived from five decades of immune research with evolutionary principles of heat tolerance. This framework offers a strong foundation for predicting the health and disease consequences of immune vulnerability to heat in a rapidly warming world.

## Introduction

Anthropogenic climate change is causing rapid increases in ambient temperatures in many parts of the globe [1, 2]. Exposure to high temperatures is predicted to be a particularly salient consequence of climate change because species have evolved thermal tolerances that were advantageous under historical environmental conditions [3, 4]. As a result, rapid warming exposes species to heat beyond their evolved upper thermal tolerance, generating a mismatch between historical thermal adaptations and present-day environmental exposures. Heat stress occurs when heat load exceeds the capacity for heat dissipation, triggering behavioral and physiological stress responses aimed at restoring a normal body temperature [5, 6]. Therefore, throughout this paper, heat stress refers specifically to elevations in body temperature that elicit compensatory responses to dissipate thermal load. Although other environmental factors, such as humidity and air flow, can affect heat load [7], here we focus on ambient temperature as the primary driver of heat stress. Extended periods of elevated body temperature can disrupt molecular and system-level processes [8, 9], and, under extreme conditions, lead to tissue damage, organ failure, and death [10]. Consequently, rising temperatures due to climate change pose substantial risks to the fitness and survival of organisms. Understanding how evolved thermal tolerance shapes vulnerability to warming is therefore critical for predicting responses to climate change.

The immune system may be particularly vulnerable to thermal mismatches under climate change because many immune processes are highly responsive to temperature [11, 12]. Transient elevations in body temperature can enhance immune function by promoting inflammatory responses and improving pathogen response, which is often considered beneficial, particularly in response to infection [13]. In contrast, sustained or extreme heat stress frequently impairs immunity. Chronic heat stress is associated with immunosuppression and increased disease risk [14, 15], and extreme heat can trigger excessive, non-specific inflammation that may be damaging [16]. However, most research examining the effects of elevated temperatures on immune function has focused on fever [17], exertional exercise [18], or occupational heat exposure [19]. Relatively little is known about how heat stress encountered during daily life influences immune function, particularly in humans. Moreover, the existing studies have rarely been interpreted through an evolutionary lens that considers how immune responsiveness to heat may be shaped by evolved thermal tolerance.

Here, we explore the immunological consequences of rising ambient temperatures driven by climate change and their implications for health and disease risk. We first review the evolutionary basis of and the physiological constraints on heat tolerance. For readers unfamiliar with evolutionary terminology, key concepts used throughout this review are defined in Box 1. We then propose that immune responsiveness to heat arises from two interdependent mechanisms: (1) the effects of temperature on metabolism and its implications for immune efficacy, and (2) changes to immune regulation via stress signaling. We show how these mechanisms are beneficial within evolved thermal ranges but may cause immune dysregulation when temperatures exceed them. We argue that immune dysregulation from heat stress has two distinct consequences –recurrent sterile inflammation and chronic immunosuppression– and examine their implications for health and disease risk. Finally, we highlight the utility of wild animal systems as models for quantifying the impacts of rising ambient temperatures on immunity. Our approach integrates more than five decades of empirical work on the relationship between immunity and temperature with evolutionary principles of heat tolerance, providing a framework for predicting the immunological consequences of climate change.

Box 1. Evolutionary concepts relevant to heat tolerance

**Figure:**
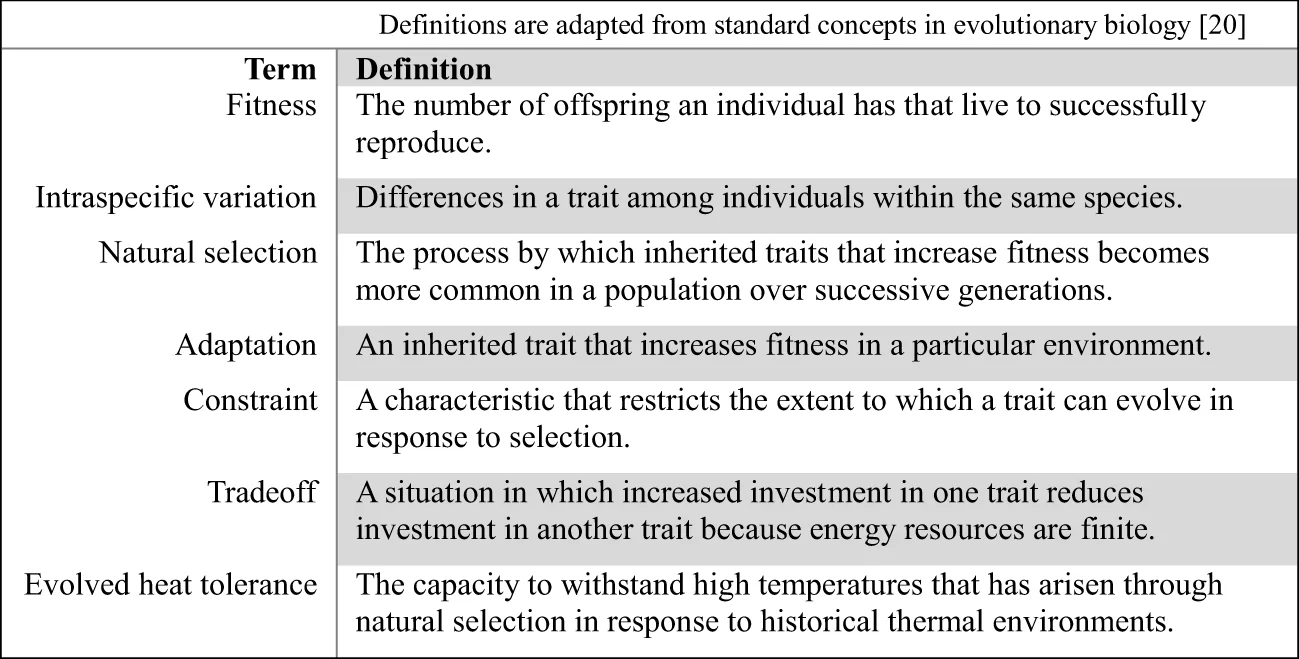

## Mechanisms underlying heat tolerance

Species vary widely in their thermal tolerances. For example, thermal tolerance often follows a latitudinal gradient, with broader tolerance ranges observed in species at high latitudes where there is greater seasonal variation in temperature [3, 21]. Such patterns indicate that different thermal environments impose distinct selective pressures [22], leading to the evolution of tolerance to temperatures most relevant to a specific environment. Importantly, there is intraspecific variation in thermal tolerance [23, 24], and this variation can be inherited [25, 26], allowing populations to evolve in response to thermal environments through natural selection. Although much of the empirical evidence for thermal adaptation comes from ectotherms, similar evolutionary processes occur in endotherms because thermoregulatory mechanisms cannot completely buffer individuals from thermal stress.

While both ends of the thermal tolerance range can evolve, the physiological consequences of heat stress differ fundamentally from cold stress. Cold exposure primarily slows metabolic processes [8], while heat more directly threatens cellular structure and function. Heat stress can destabilize cellular membranes [27], disrupt mitochondrial function [28], and denature proteins, disrupting proteostasis [29]. Disruption of proteostasis is a major consequence of heat stress, because proper protein folding underlies nearly all cellular processes and directly influences survival, longevity, and reproductive success. Consequently, heat stress is expected to impose especially strong selective pressures on mechanisms that confer tolerance to heat.

The cellular heat shock response helps preserve proteostasis during heat exposure. The accumulation of denatured and aggregated proteins activates a group of molecular chaperones, heat shock proteins (HSPs), that refold proteins or degrade irreversibly damage ones [30, 31]. Under normal conditions, HSPs bind to and repress heat shock transcription factors (HSFs). An increase in damaged proteins recruits HSPs, releasing HSFs to trimerize and rapidly (within minutes) activate HSP gene expression [32]. The resultant HSPs are classified into families based on their molecular weights; large heat shock proteins (e.g., HSP90, HSP70) actively refold or target damaged proteins, and small HSPs (typically < 30 kDa) stabilize unfolded proteins and prevent aggregation [30, 33]. This response is conserved across evolutionary lineages, with genes regulating HSP expression present across all taxa and cell types studied to date. Its presence in eukaryotes, archaea, and eubacteria indicates that the heat-shock response originated in single-celled organisms more than 2.5 billion years ago [34]. This extreme conservation reflects a universal cellular vulnerability to protein misfolding, with heat likely acting as a major selective pressure due to its destabilizing effects on protein structure.

However, the heat shock response cannot indefinitely extend heat tolerance because it is costly for cells [33, 35]. HSPs suppress general protein synthesis [36], and the chaperone functions of large HSPs are also energetically costly because they require ATP [31]. These costs have been demonstrated in *Drosophila* larvae, where more severe heat stress, eliciting higher HSP70 expression than milder heat stress, reduced both cell growth rates [37] and larval survival [38]. The tradeoff between the immediate benefits and downstream costs of the heat shock response limits it to conferring only transient heat tolerance, providing time for behavioral adjustments and physiological mechanisms to reduce thermal load. However, the extreme evolutionary conservation of the heat shock response suggests that its benefits outweigh its costs, likely because it confers an immediate survival advantage during periods of cellular stress [38]. Together, these patterns reflect strong selection on cellular mechanisms that confer heat tolerance and emphasize some of the negative consequences of exposure to heat, especially at temperatures that exceed evolved thermal limits.

Despite the consequences of heat stress for cells, the evolution of heat tolerance appears to be tightly constrained. Across species, there is far greater variation in cold tolerance than in heat tolerance [21, 39], with most eukaryotes unable to tolerate temperatures above ~ 47 °C [40]. This raises the question: if heat stress imposes strong selective pressures, what prevents the evolution of greater tolerance to heat? Two non-mutually exclusive hypotheses have been proposed to explain why upper thermal limits are similar across species: (1) species experience similar selective pressures in hot environments, and (2) heat tolerance cannot evolve beyond a certain threshold because fundamental physiological processes impose limits on the temperatures organisms can withstand [4, 39]. There is some support for the first explanation, as the cellular consequences of heat are so severe that even infrequent exposures to high temperatures may impose strong selective pressure, particularly when such exposure results in death [22]. However, growing evidence also supports the latter explanation, as fundamental processes at both systems and molecular levels operate optimally within a specific thermal range.

### Physiology constrains heat tolerance

At the organismal level, heat tolerance is shaped by the capacity of oxygen delivery to meet increasing metabolic demand. Rising temperatures accelerate metabolic rate and increase oxygen demand. However, the capacity for oxygen delivery through the respiratory and circulatory systems is limited, leading to a mismatch at high temperatures where oxygen demand exceeds supply [41, 42]. As a result, aerobic metabolism becomes constrained, forcing increased reliance on anaerobic pathways that yield less energy and cannot be sustained for prolonged periods. The shift to anaerobic pathways should impose a physiological constraint on heat tolerance because multicellular animals, particularly metazoans, depend on oxygen-based metabolism to support growth, reproduction, and maintenance [43]. Consistent with these effects, empirical work shows that reductions in cardiac rate and respiratory capacity at high temperatures coincide with declines in growth, reproduction, and population abundance in fish [44]. While this framework was developed primarily from studies of ectotherms, particularly fish and marine invertebrates [40], the underlying relationship between oxygen supply and demand is inherent to metazoans. Therefore, limits on oxygen delivery are expected to constrain heat tolerance broadly across taxa, including humans.

Thermoregulatory responses that facilitate heat dissipation, including peripheral vasodilation and redistribution of blood flow to the body surface, may further exacerbate the mismatch between oxygen supply and demand [45]. The resulting reduction in perfusion of internal organs can limit oxygen delivery, promoting local hypoxia, tissue dysfunction, accumulation of metabolic byproducts, and organ damage. For example, in mammals, heat stress is well known to reduce gastrointestinal perfusion and increase free radical production, which damages the epithelium and increases intestinal permeability [46, 47]. Limits on oxygen delivery are argued to represent the primary constraint on heat tolerance in metazoans, because oxygen depletion is expected to occur before the onset of direct cellular damage from heat exposure [40].

At the molecular level, heat tolerance is shaped by an inherent tradeoff between enzyme structural stability and flexibility [48]. As temperature increases, molecular motion accelerates, and bonds become more flexible, enhancing enzymatic reaction rates. In contrast, at low temperatures, enzymes become too rigid to efficiently catalyze reactions. The positive relationship between heat and reaction rate is finite: beyond a critical threshold, heat destabilizes enzyme structure, leading to denaturation and loss of function [8]. As a result, enzyme function is constrained to a relatively narrow thermal range. Selection for greater heat tolerance may extend function at high temperatures [49], but this typically comes at the cost of reduced efficiency at cooler temperatures that organisms are more likely to experience [8]. These inherent thermodynamic properties of enzymes impose a fundamental limit on the evolution of heat tolerance, as enzymes must maintain sufficient reaction rates to sustain metabolic function across the range of temperatures most frequently experienced.

### Heat constrains biological performance

**Fig. 1:**
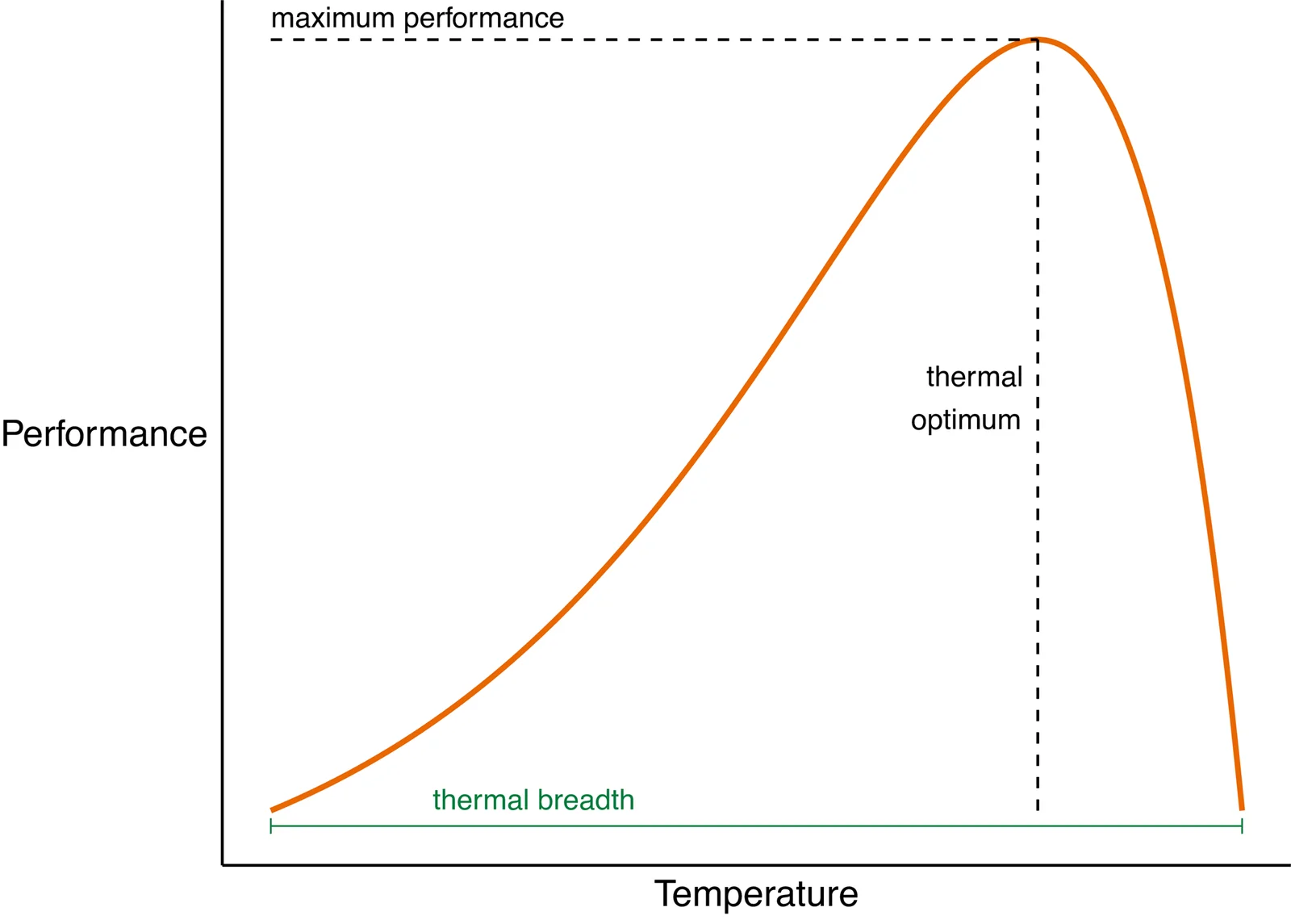
Schematic of a thermal performance curve (TPC). Temperature is shown on the x-axis and the performance (i.e., rate or efficacy) of a biological process on the y-axis. The dashed horizontal line denotes the maximum attainable performance, and the dashed vertical line indicates the temperature at which maximum performance is achieved (the thermal optimum). The green horizontal line marks the range of temperatures over which performance is greater than zero, referred to as the thermal breadth. Performance increases at low to moderate temperatures as metabolic reaction rates rise, whereas the steep decline beyond the thermal optimum reflects the loss of molecular stability at high temperatures

The thermodynamic properties of enzymes give rise to a predictable relationship between temperature and the rate or efficacy (i.e., performance) of a biological process. This relationship, known as the thermal performance curve (TPC), describes how the performance of a biological process changes across a temperature range [50]. On the cooler side of the curve, biological performance increases gradually to a peak, followed by a steep decline at higher temperatures, producing an asymmetric curve (Fig. 1). The temperature at which performance is maximized is the thermal optimum, while the thermal breath is the range between the minimum and maximum temperature where performance approaches zero [51]. TPCs are ubiquitous across different levels of biological organization and have been documented for diverse processes such as rates of photosynthesis, running speed, and population growth across a wide range of taxa, including eukaryotes, invertebrates, and vertebrates [50]. The recurrence of this curve shape across disparate processes reflects the fundamental tradeoff between enzyme flexibility and stability with temperature [52]. The rise in performance at low to moderate temperatures is driven by increased molecular motion and reaction rates that accelerate the rate of energy-dependent processes, whereas the rapid decline beyond the thermal optimum reflects systemic failure caused by molecular instability. The abrupt collapse on the warm side of the curve emphasizes the shared molecular constraints on heat tolerance and the inherent sensitivity of biological processes to temperature [40].

Variation in thermal optima and breadth among species reflects differences in evolved heat tolerance, shaped in part by their unique thermal environments. For example, tropical species tend to exhibit higher thermal optima but narrower thermal breadths than temperate species, consistent with adaptation to a narrower range of high temperatures characteristic of the tropics [4, 53]. Species-specific behavioral or physiological adaptations can also influence the parameters of their thermal performance curves. For example, physiological thermoregulation broadens thermal breadth by buffering internal body temperature from ambient conditions, increasing the range of environmental temperatures a species can tolerate [54]. Conversely, constraints on oxygen delivery in multicellular metazoans could shift performance curves relative to those of unicellular organisms [40]. Together, these lines of evidence demonstrate that while mechanisms conferring heat tolerance have evolved, upper thermal limits are also fundamentally constrained by molecular and physiological trade-offs.

### Climate change challenges evolved heat tolerance

Since thermal tolerance evolves to match the range of temperatures characteristic of an organism’s environment [3, 4, 21], rapid climate warming creates an evolutionary mismatch that exposes species to temperatures that may approach or exceed their evolved heat tolerance. As described above, heat stress can lead to physiological dysfunction through whole-body constraints on oxygen delivery [40] and compromised enzyme function [8] that affects the performance of biological processes [52]. Consistent with these expectations, climate warming has driven widespread geographic range shifts, with many species moving toward higher latitudes or elevations to track suitable thermal environments [55]. Empirical evidence for range shifts spans diverse taxa from insects [56] to mammals [57]. However, the capacity to track shifting climates is limited, and in many species, dispersal ability is constrained by geographic barriers, ecological suitability, and habitat disturbance [58]. This increases the likelihood of chronic or repeated exposure to high temperatures that species did not evolve to tolerate.

In the remainder of this article, we expand on how a growing evolutionary mismatch between evolved heat tolerance and rising ambient temperatures will impact the immune system. We consider two primary mechanisms that influence immune responsiveness to heat: (1) thermal performance effects on immunity, and (2) changes to immune regulation via signals of stress. We discuss contexts in which these mechanisms are beneficial within evolved thermal ranges but may lead to immune dysregulation from heat stress under climate change.

## Thermal performance properties influence immune responsiveness to heat

Immune responses depend on enzyme-mediated reactions and are therefore expected to exhibit thermal performance relationships similar to other biological processes. We define immune performance as the capacity of the immune system to mount an effective response to a challenge [59], which is commonly assessed using functional assays such as bacterial killing ability or lytic capacity [60]. A moderate increase in temperature should enhance immune performance by increasing metabolic rates, resulting in more available energy for processes such as phagocytosis, cytotoxic activity, and the production of effector molecules. This relationship will persist until reaching a thermal optimum, beyond which elevated temperatures disrupt protein stability, membrane integrity, and cellular metabolism, causing a rapid decline in immune performance (Fig. 1). Therefore, thermal performance properties enhance immunity under some conditions, but also render the immune system vulnerable to dysregulation when heat exposures exceed evolved thermal tolerance. Importantly, this relationship arises from the thermodynamic properties of enzymes rather than changes to immune regulation.

In line with these predictions, immune thermal performance curves have been documented across a wide range of taxa. Such relationships are well characterized in ectothermic species, including reptiles [61], amphibians [62, 63], and insects [64], where body temperature closely reflects ambient conditions. However, immune thermal performance curves have also been observed in endotherms, including hamsters [65], voles [66], and primates [67], demonstrating that high ambient temperatures can also affect the immunity of species with physiological thermoregulation. Differences in immune thermal performance curves among species likely reflect variation in evolved heat tolerance. For example, immune performance in ectotherms often peaks at relatively low temperatures (approximately 18–25 °C) [62–64], whereas studies in endotherms suggest that immune performance peaks at higher temperatures, around 30 °C [65–67]. Higher thermal optima in endotherms likely reflect the ability of physiological thermoregulation to increase tolerance to ambient conditions. Variation in immune thermal optima among species may also reflect adaptation to distinct thermal environments. For instance, immune performance declines above 25 °C in several species of *Rhinella* toads [63], yet continues to increase through 34 °C in the desert-dwelling toad *Anaxyrus punctatus* [62], consistent with its adaptation to hotter desert climates. Together, these patterns suggest that immune performance is constrained by evolved heat tolerance from adaptation to distinct thermal environments.

Some of the strongest empirical evidence for the thermal performance properties of immunity comes from in vitro experiments with human immune cells. These studies show that short-term exposure to temperatures within the human febrile range (approximately 38–41 °C) can enhance the performance of innate immune cells, including increased phagocytic activity and bacterial killing in macrophages and neutrophils, over durations of roughly 2–6 h [68, 69]. In contrast, exposure to temperatures above the febrile range (approximately 42–45 °C) rapidly impairs immune performance [70, 71] and can be lethal to cells even over short periods. For instance, exposure to temperatures between 42 and 43 °C has been shown to cause rapid cell death in dendritic cells within as little as 15 min [72]. Together, these findings illustrate a clear thermal performance relationship for human immune cells and suggest that the benefits of fever do not solely arise from direct pathogen inhibition but rather an increased immune advantage at warmer temperatures [17].

Importantly, the same thermodynamic principles apply whenever immune cells experience elevated temperatures, regardless of whether warming coincides with infection. For example, empirical studies in humans demonstrate that heat stress within the febrile range induced by exercise [18, 73] or passive heating [74] is associated with increased immune activity, evidenced by enhanced mobilization and count of circulating leukocytes. Growing mismatches between heat tolerance and rising ambient temperatures will expose immune systems to thermal conditions they did not evolve to tolerate, increasing the risk of immune dysregulation from thermal performance effects. Repeated exposure to moderate heat stress may increasingly elevate immune activity in the absence of infection, promoting recurrent sterile inflammation (i.e., inflammation in the absence of pathogens). Even though the heat shock response can transiently support increased immune performance during moderate heat exposure, it cannot indefinitely preserve cellular integrity under sustained or extreme thermal stress. As a result, prolonged or extreme heat stress that exceeds immune thermal optima may lead to periods of immunosuppression.

## Signals of stress regulate immune responses to heat

During heat stress, the effects of thermal performance on immunity do not act in isolation. The immune system is also highly responsive to signals of physiological stress, which convey information about internal challenges and help coordinate an appropriate responses [75, 76]. The immune system is regulated by a variety of molecules that signal physiological stress, but here we focus on two classes of signals that are reliably elicited by heat stress. These are (1) damage-associated molecular patterns (DAMPs), which are endogenous indicators of cellular damage [77], and (2) neuroendocrine signals that reflect systemic physiological stress [75]. Immune responsiveness to stress signals is typically beneficial because it helps regulate immune function to match the nature and severity of a challenge. However, climate change is expected to alter the intensity, duration, and frequency of heat-induced stress signaling, potentially increasing the risk of immune dysregulation. We expect the nature of this dysregulation to depend on the duration and severity of the heat stress because it will elicit different types of stress signals. In the following sections, we consider the roles of DAMPs and neuroendocrine signaling in regulating immune responses to heat stress.

### Heat stress generates damage-associated molecular pathways

Mammalian DAMPs promote rapid, localized immune responses to tissue damage and are important for initiating sterile inflammation required for tissue repair [76]. DAMPs comprise a diverse set of endogenous molecules, including peptides, lipids, and nucleic acids, that are actively excreted by stressed cells or passively released during cell death. Extracellular DAMPs stimulate innate immune receptors, increasing proinflammatory cytokine and chemokine expression, while intracellular DAMPs activate inflammasome pathways that similarly promote inflammation [77, 78]. Together, these pathways rapidly recruit leukocytes to sites of cellular damage.

Heat stress elicits predictable cellular responses that generate DAMPs and promote sterile inflammation. For example, HSPs can act as DAMPs when released extracellularly following sterile damage [79]. Studies in human cells have shown that, at high concentrations, extracellular HSP (e.g., HSP60, HSP70) bind to Toll-like receptors 2 and 4 on innate effector cells and stimulate expression of proinflammatory cytokines [80, 81]. Heat stress also promotes oxidative damage that generates DAMPs. High temperatures accelerate metabolic rate and mitochondrial respiration, increasing the production and leakage of reactive oxygen and nitrogen species (RONS) from the electron transport chain [28]. While RONS in small quantities are necessary for effective immune defense [82], RONS production that exceeds antioxidant capacity can damage cells [83]. Because RONS are generated primarily within the mitochondrial matrix, mitochondria are disproportionately vulnerable to oxidative damage, particularly their lipid membranes and DNA (mtDNA), which has reduced repair capacity compared to nuclear DNA [84]. Oxidative damage to mitochondrial membranes allows oxidized mtDNA to escape into the cytosol [85], where it can activate innate immune receptors and promote cell death [77]. Extracellular mtDNA binds to Toll-like receptor 9 on innate immune cells, increasing proinflammatory cytokine expression [84]. Cytosolic mtDNA similarly promotes inflammatory signaling by activating inflammasomes and cytosolic DNA–sensing pathways [86]. Because oxidized mtDNA is resistant to degradation, it can accumulate in the cytosol and prolong immune activation [84, 87]. Importantly, mtDNA damage further impairs mitochondrial respiration, exacerbating RONS production and reinforcing this cycle [88].

#### Heat-driven DAMPs cause sterile inflammation

Studies of heat stroke in humans provide evidence that heat-driven DAMPs can trigger sterile inflammation [16]. Heat stroke is a severe form of heat stress typically defined by prolonged elevations in core body temperature, often exceeding ~ 40 °C [10]. It elicits a massive systemic inflammatory response, the magnitude of which closely resembles that observed in sepsis. Excessive inflammatory activation during heat stroke can result in widespread tissue injury and contribute to multi-organ dysfunction [10]. Although heat stroke represents an extreme pathological response to heat exposure, even moderate heat stress may generate sufficient DAMPs to promote sterile inflammation on a much smaller scale. Studies in humans show that elevated core temperature from intensive exercise increases extracellular mtDNA levels and inflammatory markers, such as circulating leukocytes [89, 90]. Exercise can also increase extracellular heat shock proteins that are associated with higher white blood cell counts [91, 92].

Climate change may increase the risk of immune dysregulation by increasing the frequency and intensity of DAMP signaling. As rising ambient temperatures exceed evolved thermal tolerance, heat stress is expected to become more frequent and severe, elevating the likelihood of DAMP generation and recurrent sterile inflammation. This risk may be particularly pronounced for mitochondrial DAMPs. Repeated heat exposure could promote cumulative mitochondrial dysfunction, increasing mtDNA oxidation and the probability of mtDNA release into the cytosol [28, 85]. Therefore, even if individual episodes of moderate heat stress are insufficient to elicit strong DAMP signaling, repeated heat exposure may progressively increase cytosolic mtDNA and the likelihood of engagement with intracellular innate immune sensors. In this way, thermal mismatches under climate change may convert an otherwise beneficial damage-sensing adaptation into a driver of recurrent sterile inflammation.

### Neuroendocrine signals of stress affect immune regulation

The immune system is also regulated by stress signals from the central nervous system (CNS). The CNS is sensitive to diverse stimuli, including adverse ecological or social environments and psychological distress, and uses the stress response to reallocate energy to cope with these challenges [93]. Although acute stress can transiently enhance immune activity [94], chronic stress is generally associated with immunosuppression [95]. Here, we focus on how chronic neuroendocrine signaling regulates immune function and consider its relevance for prolonged heat stress.

The CNS primarily interacts with the immune system via glucocorticoids produced by the hypothalamic pituitary (HPA) axis and catecholamines (e.g., epinephrine and norepinephrine) produced by the sympathetic-adrenal-medullary (SAM) axis. The SAM and HPA axes function as an integrated stress response in which detection of a stressor by the sympathetic nervous system induces the rapid release of catecholamines, followed by subsequent glucocorticoid secretion from the adrenal cortex [93]. Chronic secretion of neuroendocrine hormones shifts immune investment away from Th1-mediated cellular immunity and toward Th2-biased, humoral responses, which has been primarily documented in mouse models [75, 96]. Elevated circulating glucocorticoids preferentially engage the glucocorticoid receptor (GR), which is broadly expressed across immune cell types [97]. GR activation reduces the expression of proinflammatory cytokines and promotes the expression of anti-inflammatory cytokines [98]. Catecholamines reinforce this shift by activating β-adrenergic receptors expressed on Th1 cells. This suppresses Th1-associated cytokine signaling, permitting increased Th2 differentiation [96, 99]. Therefore, sustained glucocorticoid and catecholamine signaling suppresses cell-mediated immunity by limiting proinflammatory effector activity, while promoting humoral immune processes.

While suppression of non-specific inflammation during sustained stress may reduce the risk of self-inflicted tissue damage [98, 99], extensive experimental evidence shows that prolonged neuroendocrine stress signaling compromises the immune system’s ability to fight infection. For example, mice subjected to restraint stress and infected with a virus exhibit reduced virus-specific cytokine production and impaired T-cell responses [95, 100]. Importantly, blocking glucocorticoid receptors restores cytokine production and T-cell function, demonstrating that the immunosuppressive effect of glucocorticoids reduces the immune system’s ability to respond to infectious challenges [100]. In humans, chronically stressed populations show reduced immune responses to vaccination, a useful proxy for increased infection risk because it reflects a diminished ability to mount effective responses to immune challenges [95]. Additional work demonstrates that individuals who report sustained psychological distress are more likely to develop illness following inoculation with a rhinovirus [101]. Heat stress activates the same neuroendocrine pathways as other stressors [6, 102]; therefore, these mechanisms provide a plausible framework for understanding how chronic heat exposure may influence immune regulation.

#### Chronic heat stress causes immunosuppression

Direct evidence linking chronic neuroendocrine signaling to heat-driven immunosuppression remains limited. However, studies in laboratory animals, livestock, and humans consistently demonstrate that chronic heat exposure suppresses immune function. For example, mice exposed to repeated heat stress (e.g., daily exposure to 38 °C for several hours) show reduced immune responsiveness to vaccination [103, 104]. An extensive body of work in livestock [14, 105] shows that exposure to elevated ambient temperatures for extended periods, typically 2–7 weeks at approximately 30–40 °C depending on species, consistently results in broad suppression of immune function, including reductions in leukocyte counts and inflammatory signaling [106, 107]. Evidence from humans subject to chronic heat exposure from their occupations similarly shows reduced innate immune activity and lower white blood cell counts [19, 108]. Importantly, it is unclear whether these effects are due to chronic neuroendocrine signaling, as repeated or prolonged exposure to the same stressor can lead to physiological acclimation and reduced HPA-axis responsiveness [6, 109]. However, even if physiological acclimation occurs, it does not appear sufficient to eliminate the immunosuppressive effects of chronic heat stress.

Emerging evidence suggests that neuroendocrine signaling may contribute to heat-driven immunosuppression. For example, pigs exposed to prolonged heat stress (21 days at ~ 35 °C) show elevated circulating glucocorticoids in conjunction with reduced white blood cell counts and decreased expression of IL-2 and IFN-γ, consistent with suppression of cell-mediated immunity [110]. Therefore, while regulation of the immune system by neuroendocrine signals of stress is beneficial within evolved thermal limits, it may leave the immune system vulnerable to dysregulation when ambient temperatures exceed those limits.

## Heat-driven immune dysregulation has important implications for health and disease

**Fig. 2:**
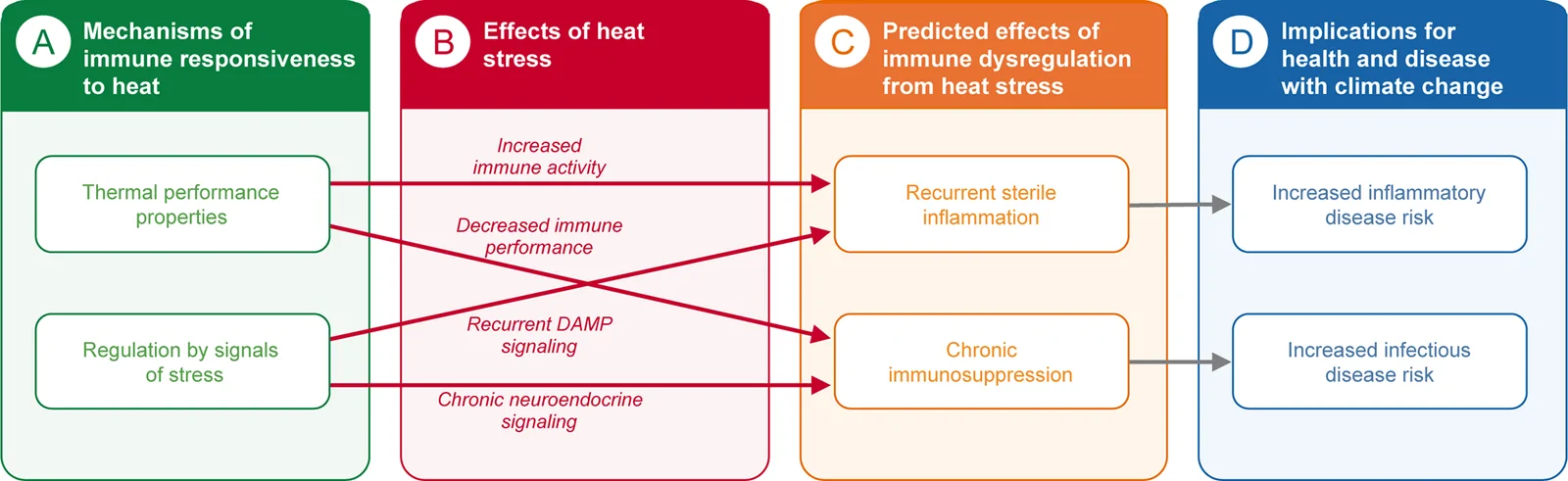
A framework for predicting the immunological consequences of climate change. Panel **A** illustrates two core mechanisms that influence immune responsiveness to heat. Panel **B** shows how these mechanisms render the immune system vulnerable to dysregulation during heat stress. Panel **C** depicts the predicted consequences of immune dysregulation from heat stress, and Panel **D** highlights the downstream health implications

We propose that thermal performance properties and signals of stress provide two complementary mechanisms through which heat influences immunity (Fig. 2A). These mechanisms can affect immunity in distinct ways depending on the severity and duration of heat stress, and growing thermal mismatches under climate change may promote recurrent sterile inflammation and chronic immunosuppression (Fig. 2B & C). Importantly, these mechanisms are not mutually exclusive and likely interact during heat stress. For example, immunosuppression during chronic heat stress may reflect both neuroendocrine suppression of immune activity and constraints on sustaining cellular heat tolerance. Disentangling their relative contributions will remain challenging, but experimental approaches may help isolate their individual effects. In vitro cell culture can isolate thermal performance effects [59], whereas animal models with disrupted stress signaling pathways (e.g., glucocorticoid receptor knockouts) may help isolate regulation by stress signals [100]. However, because both mechanisms occur simultaneously during heat exposure, understanding their interaction will be equally important for predicting immune dysregulation under climate change. In the following sections, we consider the distinct health implications of sterile inflammation and immunosuppression in the context of climate change (Fig. 2D).

### Sterile inflammation increases inflammatory disease risk

We expect climate warming to increase sterile inflammation through both thermal performance effects and DAMP signaling (Fig. 2). Although DAMPs are beneficial for tissue repair, recurrent or unresolved signaling contributes to chronic inflammation implicated in a wide range of diseases [e.g., cardiovascular, neurodegenerative, and autoimmune disorders [78, 111] ]. The DAMPs we predicted to be most relevant to heat stress, HSPs, and mtDNA, have been directly linked to inflammatory pathology. Studies in humans have shown that HSP70 and HSP96 are elevated in rheumatoid arthritis and promote inflammation through TLR activation of innate immune cells [112, 113], while oxidized mtDNA drives excessive type I interferon production in systemic lupus erythematosus [114]. Importantly, these are selected examples of how DAMPs that can be generated during heat stress contribute to inflammatory pathology, and similar mechanisms are likely relevant to many other diseases [e.g., asthma and cancer [78, 111] ]. Although direct evidence linking recurrent heat exposure, DAMP signaling, and inflammatory disease remains limited, these findings suggest a plausible mechanism by which repeated heat stress could contribute to disease development or severity.

Heat-driven DAMPs may also contribute to age-associated inflammatory pathology. Across species, aging is characterized by chronic, low-grade inflammation [i.e., inflammaging [115] ], which increases the risks of non-communicable diseases such as cancer, cardiovascular disease, diabetes, and neurodegenerative disorders [116]. Endogenous danger signals are thought to play a key role in driving inflammaging, and mtDNA is likely central to this process. mtDNA mutations accumulate with age, leading to impaired respiratory function, increased RONS production, and cellular senescence [84, 117]. Consistent with this, circulating levels of extracellular mtDNA increase with age in humans and are associated with elevated proinflammatory cytokines [118]. Heat stress may accelerate these processes because high temperatures similarly increase oxidative mitochondrial damage [28], and heat exposure has already been associated with accelerated biological aging in humans [119]. Together, these findings suggest that rising temperatures could promote inflammatory processes implicated in aging via heat-driven DAMP signaling, with consequences for the risk of developing age-related diseases.

Heat-driven sterile inflammation could also cause dysbiosis of the gut microbiome, defined as alterations in microbial composition and metabolic function that disrupt community homeostasis. Inflammation alters the intestinal environment by increasing epithelial oxygenation and RONS production. These conditions are poorly tolerated by obligate anaerobes but favor the expansion of aerobic and facultatively anaerobic taxa with more metabolic flexibility [120]. This shift away from obligate anaerobes toward oxygen-tolerant groups, particularly Proteobacteria, is a hallmark of dysbiosis and reflects the ability of these taxa to exploit inflamed environments [121]. Consistent with this mechanism, elevated temperatures are associated with declines in anaerobe-dominated phyla such as Firmicutes and increases in Proteobacteria across diverse host species [122]. These shifts can disrupt beneficial host–microbiome interactions. For example, obligate anaerobes ferment dietary fiber and produce short-chain fatty acids, which serve as important energy sources for the host and are key immunomodulators [123, 124]. In contrast, expansion of Proteobacteria is often associated with inflammatory bowel disease in humans, and includes many known gut pathogens, such as *Escherichia coli*, *Klebsiella* spp., and *Proteus* spp [125]. Together, these findings suggest that heat-driven sterile inflammation may indirectly increase disease risk by promoting dysbiosis of the gut microbiome.

As outlined here, dysregulated sterile inflammation has clear implications for inflammatory disease risk and may also adversely affect health by accelerating aging processes and disrupting the gut microbiome. Further research is needed to quantify the extent to which heat-driven sterile inflammation contributes to these outcomes, including how the magnitude and frequency of heat stress shape inflammatory pathology.

### Heat-induced immunosuppression will increase infectious disease risk

**Fig. 3:**
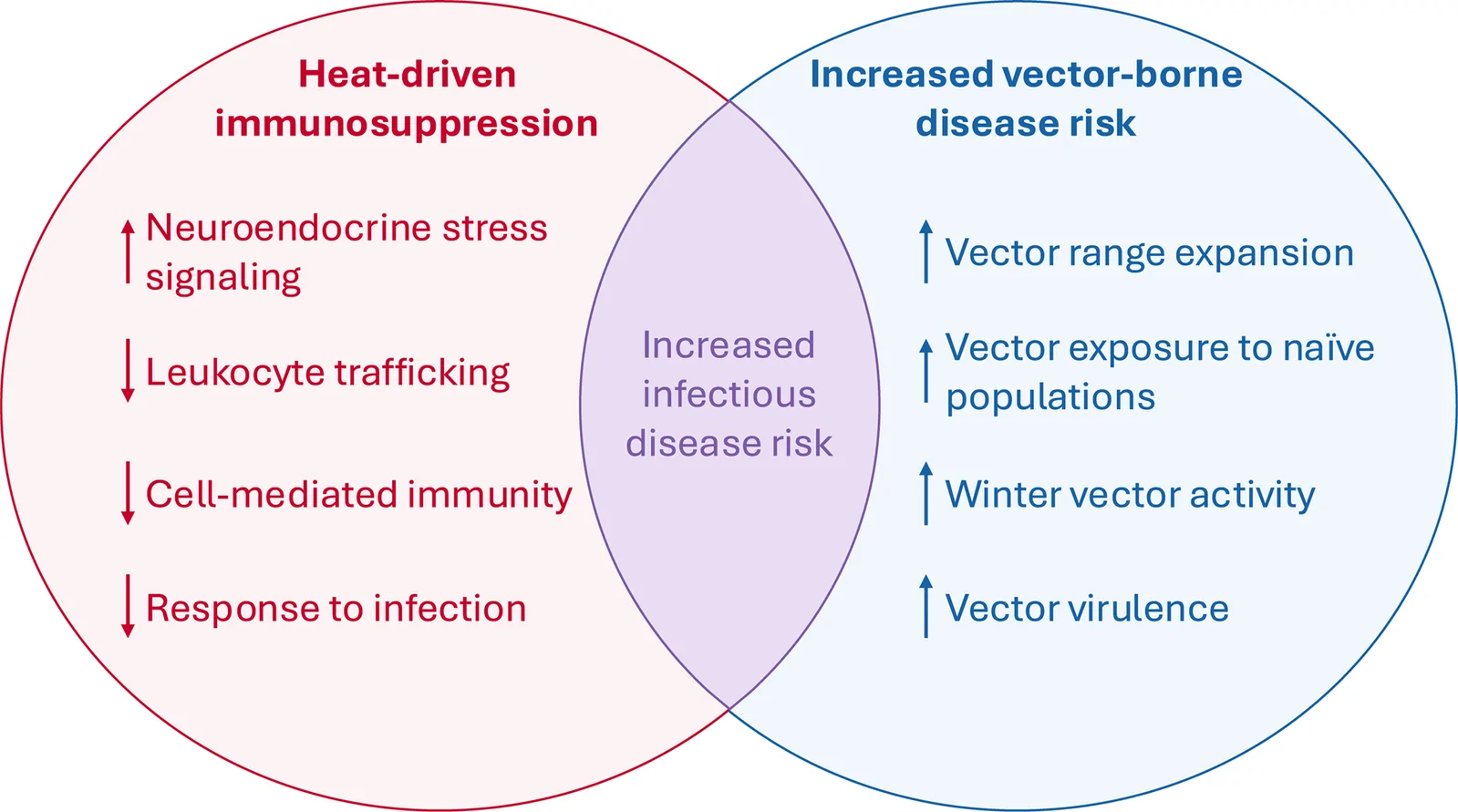
Overlapping drivers of infectious disease risk under climate warming. The red circle summarizes the consequences of heat-driven immunosuppression, while the blue circle shows climate-driven changes in vector activity. Their overlap illustrates that rising infectious disease risk results from both reduced host resistance to infection and increased exposure to more virulent disease vectors

We predict that chronic heat stress will increase infectious disease risk through sustained neuroendocrine stress signaling and thermal constraints on immunity (Fig. 2). Consistent with this prediction, evidence from humans, mice, and livestock indicates that chronic stress and prolonged heat exposure are associated with immunosuppression [95, 105]. Climate change is also predicted to increase infectious disease risk by altering host–pathogen interactions. Warming temperatures are expected to shift the geographic ranges of infectious disease vectors. Empirical evidence already shows marked expansion of vector species and increased prevalence of vector-borne diseases in humans, including tick-borne encephalitis [126] and mosquito-borne diseases such as West Nile virus, Chikungunya, and dengue [127]. Range expansion is particularly concerning because it exposes immunologically naïve populations to novel pathogens, a risk exacerbated by immunosuppression. At the same time, rising temperatures will facilitate vector and pathogen survival during historically cold seasons, removing an important constraint on population growth [128]. Consistent with this prediction, warming has been associated with increased frequency and severity of mosquito-borne illness in humans [129]. We expect these changes, combined with immunosuppression from chronic heat exposure, to substantially increase infectious disease burden under climate change (Fig. 3).

Heat-driven immunosuppression may disproportionately increase vulnerability to infectious disease in younger individuals. Early-life immunity relies more heavily on innate defenses, while adaptive, antigen-specific responses develop gradually with cumulative pathogen exposure [130]. Sustained glucocorticoid signaling is therefore particularly problematic because it suppresses the nonspecific inflammatory responses that are critical in early life. This has led to speculation that the immune-related effects of climate change will disproportionately affect young individuals relative to other populations [67, 131].

Beyond increasing susceptibility to novel infections, immunosuppression may also impair the control of existing infections. Many viruses can remain in a latent state for extended periods during which hosts remain asymptomatic. Stress has been shown to increase the likelihood of reactivation of these infections and has been studied extensively in the context of herpes simplex virus (HSV). For example, experimentally stressed mice exhibit greater reactivation of HSV [132] caused by neuroendocrine suppression of HSV-specific cytotoxic T-cell responses [133]. Consistent with these findings, humans who report chronic psychological stress have more frequent recurrences of genital herpes [134]. Together, this evidence suggests that immunosuppression from chronic heat stress may increase the reactivation of latent infections under climate change.

Similarly, stress-induced immunosuppression also impairs wound healing. Mice subjected to restraint stress exhibit significantly delayed wound closure, but this effect is reversed by blocking glucocorticoid receptors [135]. Congruent patterns have been observed in humans, where individuals experiencing chronic psychological stress show slower wound healing [136]. Chronic heat exposure produces similar effects, where mice subjected to chronic heat stress (i.e., 43 °C for two weeks) showed delayed wound healing [137]. Slower wound healing may, in turn, increase the risk of secondary infections with climate change [138].

As outlined here, immunosuppression has direct consequences for infection risk, particularly for young individuals, and may also indirectly exacerbate disease burden by slowing wound healing. When combined with the increasing virulence of vector species, immunosuppression could further increase infectious disease risk under climate change (Fig. 3). Future research is needed to quantify how heat-induced immunosuppression influences infection rates and disease severity. This type of research is critical for improving predictions of public health risks posed by climate warming.

## Studies of wild animals can help evaluate the immunological consequences of climate change

Most research on the relationship between heat and immunity has been conducted under controlled experimental conditions. This work is essential for quantifying the effects of temperature on immune function and for identifying the mechanisms through which heat influences immunity [11, 12]. However, experimental studies necessarily simplify the conditions under which heat is experienced in order to isolate its effects [139]. As a result, heat stress experiments cannot fully replicate the spatial, temporal, and behavioral complexity of heat exposure in natural environments. Changes in weather conditions combined with variation in microclimate use make it difficult to quantify the temperatures experienced by individuals in the wild [140], and many species possess behavioral adaptations for dissipating heat load that can be difficult to recreate under experimental conditions [141, 142]. Consequently, controlled experiments alone may not fully capture how immune systems respond to heat under natural conditions.

Wild animal models are an important complement to experimental approaches for evaluating the immunological consequences of climate change. Studying immunity in wild animals allows direct observation of how immune systems respond to naturally occurring variation in ambient temperature. Wild systems also inherently include key sources of variation that shape heat tolerance, such as life-history stage, nutritional state, prior thermal exposure, and thermoregulatory behavior [51, 141]. As a result, studying wild systems offers an opportunity to evaluate whether mechanisms identified under controlled conditions translate into meaningful health outcomes under environmental conditions in which climate warming is actually experienced. This perspective is particularly relevant because humans, like wild animals, experience heat as a part of a complex suite of environmental and behavioral factors rather than as an isolated stressor. Leveraging established long-term field studies of wild animal populations, particularly primates, allows researchers to track the health consequences of heat exposure over decades while simultaneously measuring environmental, behavioral, and physiological variables that can be difficult to quantify consistently in human populations [143, 144].

Fortunately, biomarkers such as neopterin and soluble urokinase plasminogen activator receptor (suPAR) have expanded opportunities to study immune activation in wild animals. Because these biomarkers can be measured from urine and incorporated into existing long-term field studies, they allow for non-invasive immune monitoring with minimal disturbance to study populations. Neopterin reflects IFN-γ–mediated macrophage activation and has been used to assess temperature-driven variation in cell-mediated immunity [67, 145]. In contrast, suPAR indicates broad inflammatory activation and is elevated during sterile tissue damage [146], making it useful to measure inflammation from heat exposure. Importantly, non-invasive sampling allows more frequent measurement of immune biomarkers, making it possible to identify the temperature ranges associated with meaningful changes in immunity under natural conditions [67]. Therefore, incorporating perspectives from wild systems can assist in generating accurate predictions of the immunological consequences and associated health risks of our warming climate.

## Conclusion

In this review, we develop an evolutionary framework for predicting the immunological consequences of climate change by interpreting extensive evidence for immune responsiveness to heat in the context of evolved thermal tolerance. Although the effects of temperature on immune processes have been studied for decades, they have rarely been considered from this perspective. By bridging these literatures, we show that immune responses to heat arise through two broad mechanisms: the effects of thermal performance properties on immunity and regulation of immune function via signals of stress. While both mechanisms can be beneficial within evolved thermal tolerance, they also create vulnerability to immune dysregulation when environmental temperatures exceed upper thermal limits. Our framework also helps reconcile opposing immune responses to heat by showing how these mechanisms produce different effects on immunity depending on the intensity and duration of heat exposure. However, predicting immune dysregulation under climate change is challenging because real-world heat exposures are shaped by ecological, physiological, and behavioral contexts that are difficult to replicate experimentally. As a result, studies of wild populations, combined with an evolutionary perspective on thermal tolerance, provide an important complement to experimental research for predicting the immunological consequences of an increasingly warming climate.

## Data Availability

No datasets were generated or analysed during the current study.

## References

[1] Cook J, Nuccitelli D, Green SA, Richardson M, Winkler B et al (2013) Quantifying the consensus on anthropogenic global warming in the scientific literature. Environ Res Lett 8(2):024024

[2] IPCC (2023) Summary for policymakers. In Climate Change 2021: The Physical Science Basis. Contribution of Working Group I to the Sixth Assessment Report of the Intergovernmental Panel on Climate Change. Cambridge Univ. Press, Cambridge, UK/New York

[3] Sunday JM, Bates AE, Dulvy NK (2010) Global analysis of thermal tolerance and latitude in ectotherms. Proc Biol Sci 278(1713):1823–1830 10.1098/rspb.2010.1295 PMC3097822 21106582

[4] Bennett JM, Sunday J, Calosi P, Villalobos F, Martínez B et al (2021) The evolution of critical thermal limits of life on Earth. Nat Commun 12(1):1198 10.1038/s41467-021-21263-8 PMC7895938 33608528

[5] Horowitz M (2002) From molecular and cellular to integrative heat defense during exposure to chronic heat. Comp Biochem Physiol Mol Integr Physiol 131(3):475–483 10.1016/s1095-6433(01)00500-1 11867273

[6] Bernabucci U, Lacetera N, Baumgard LH, Rhoads RP, Ronchi B, Nardone A (2010) Metabolic and hormonal acclimation to heat stress in domesticated ruminants. Animal 4(7):1167–1183 10.1017/S175173111000090X 22444615

[7] Renaudeau D, Collin A, Yahav S, De Basilio V, Gourdine JL, Collier RJ (2012) Adaptation to hot climate and strategies to alleviate heat stress in livestock production. Animal 6(5):707–728 10.1017/S1751731111002448 22558920

[8] Fields PA (2001) Review: Protein function at thermal extremes: balancing stability and flexibility. Comp Biochem Physiol Mol Integr Physiol 129(2–3):417–431 10.1016/s1095-6433(00)00359-7 11423314

[9] Pörtner HO (2002) Climate variations and the physiological basis of temperature dependent biogeography: systemic to molecular hierarchy of thermal tolerance in animals. Comp Biochem Physiol Mol Integr Physiol 132(4):739–761 10.1016/s1095-6433(02)00045-4 12095860

[10] Yan Y-E, Zhao Y-Q, Wang H, Fan M (2006) Pathophysiological factors underlying heatstroke. Med Hypotheses 67(3):609–617 10.1016/j.mehy.2005.12.048 16631316

[11] Evans SS, Repasky EA, Fisher DT (2015) Fever and the thermal regulation of immunity: the immune system feels the heat. Nat Rev Immunol 15(6):335–349 10.1038/nri3843 PMC4786079 25976513

[12] Maloney E, Duffy D (2024) Deciphering the relationship between temperature and immunity. Discov Immunol 3:1–13 10.1093/discim/kyae001 PMC10917241 38567294

[13] Hasday JD, Fairchild KD, Shanholtz C (2000) The role of fever in the infected host. Microbes Infect 2(15):1891–1904 10.1016/s1286-4579(00)01337-x 11165933

[14] Inbaraj S, Sejian V, Bagath M, Bhatta R (2016) Impact of Heat Stress on Immune Responses of Livestock: A Review. Pertanika J Trop Agric Sci 39(4):459–482

[15] Chauhan SS, Rashamol VP, Bagath M, Veerasamy Sejian, Dunshea FR (2021) Impacts of heat stress on immune responses and oxidative stress in farm animals and nutritional strategies for amelioration. Int J Biometeorol 65(7):1231–1244 10.1007/s00484-021-02083-3 33496873

[16] Iba T, Helms J, Nagaoka I, Kondo Y, Ferrer R, Levy JH (2025) Sepsis and heatstroke: overlapping and distinct mechanisms of systemic inflammation. Inflamm Res 74(1):173 10.1007/s00011-025-02145-x 41296074

[17] Hasday JD, Singh IS (2000) Fever and the heat shock response: distinct, partially overlapping processes. Cell Stress Chaperones 5(5):471–480 10.1379/1466-1268(2000)005<0471:fathsr>2.0.co;2 PMC312879 11189454

[18] Peake J, Peiffer JJ, Abbiss CR, Nosaka K, Okutsu M et al (2008) Body temperature and its effect on leukocyte mobilization, cytokines and markers of neutrophil activation during and after exercise. Eur J Appl Physiol 102(4):391–401 10.1007/s00421-007-0598-1 17962974

[19] Jafari MJ, Pirposhteh EA, Dehghan SF, Khodakarim S, Jafari M (2020) Relationship between heat stress exposure and some immunological parameters among foundry workers. Int J Biometeorol 64(5):853–861 10.1007/s00484-020-01874-4 32036432

[20] Futuyma DJ, Kirkpatrick M (2023) Evolution, 5th edn. Oxford University Press, New York

[21] Addo-Bediako A, Chown SL, Gaston KJ (2000) Thermal tolerance, climatic variability and latitude. Proc Biol Sci 267(1445):739–745 10.1098/rspb.2000.1065 PMC1690610 10819141

[22] Buckley LB, Huey RB (2016) How Extreme Temperatures Impact Organisms and the Evolution of their Thermal Tolerance. Integr Comp Biol 56(1):98–109 10.1093/icb/icw004 27126981

[23] Nati JJH, Svendsen MBS, Marras S, Killen SS, Steffensen JF et al (2021) Intraspecific variation in thermal tolerance differs between tropical and temperate fishes. Sci Rep 11(1):21272 10.1038/s41598-021-00695-8 PMC8553816 34711864

[24] Sasaki M, Barley JM, Gignoux-Wolfsohn S, Hays CG, Kelly MW et al (2022) Greater evolutionary divergence of thermal limits within marine than terrestrial species. Nat Clim Chang 12(12):1175–1180

[25] Benfey TJ, Gonen S, Bartlett CB, Garber AF (2024) Thermal tolerance has high heritability in Atlantic salmon, *Salmo salar*. Aquac Rep 37:102249

[26] Hangartner S, Hoffmann AA (2016) Evolutionary potential of multiple measures of upper thermal tolerance in Drosophila melanogaster. Funct Ecol 30(3):442–452

[27] Vigh L, Nakamoto H, Landry J, Gomez-Munoz A, Harwood JL, Horvath I (2007) Membrane Regulation of the Stress Response from Prokaryotic Models to Mammalian Cells. Ann N Y Acad Sci 1113(1):40–51 10.1196/annals.1391.027 17656573

[28] Belhadj Slimen I, Taha N, Abdeljelil G, Hajer D, Moncef BM, Manef A (2014) Reactive oxygen species, heat stress and oxidative-induced mitochondrial damage. A review. Int J Hyperth 30(7):513–523 10.3109/02656736.2014.971446 25354680

[29] Ahmed K, Zaidi SF, Mati-ur-Rehman, Rehman R, Kondo T (2020) Hyperthermia and protein homeostasis: Cytoprotection and cell death. J Therm Biol 91:102615 10.1016/j.jtherbio.2020.102615 32716865

[30] Lindquist S, Craig EA (1988) The Heat-Shock Proteins. Annu Rev Genet 22:631–677 10.1146/annurev.ge.22.120188.003215 2853609

[31] Richter K, Haslbeck M, Buchner J (2010) The Heat Shock Response: Life on the Verge of Death. Mol Cell 40(2):253–266 10.1016/j.molcel.2010.10.006 20965420

[32] Lindquist S (1986) The Heat-Shock Response. Annu Rev Biochem 55:1151–1191 10.1146/annurev.bi.55.070186.005443 2427013

[33] Feder ME, Hofmann GE, HEAT-SHOCK PROTEINS (1999) MOLECULAR CHAPERONES, AND THE STRESS RESPONSE: Evolutionary and Ecological Physiology. Annu Rev Physiol 61(1):243–282 10.1146/annurev.physiol.61.1.243 10099689

[34] Singh IS, Hasday JD (2013) Fever, hyperthermia and the heat shock response. Int J Hyperth 29(5):423–435 10.3109/02656736.2013.808766 23863046

[35] Sørensen JG, Kristensen TN, Loeschcke V (2003) The evolutionary and ecological role of heat shock proteins. Ecol Lett 6(11):1025–1037

[36] Duncan R, Hershey JW (1984) Heat shock-induced translational alterations in HeLa cells. Initiation factor modifications and the inhibition of translation. J Biol Chem 259(19):11882–11889 6384217

[37] Feder JH, Rossi JM, Solomon J, Solomon N, Lindquist S (1992) The consequences of expressing hsp70 in Drosophila cells at normal temperatures. Genes Dev 6(8):1402–1413 10.1101/gad.6.8.1402 1644286

[38] Krebs RA, Feder ME (1997) Deleterious consequences of Hsp70 overexpression in Drosphilla melanogaster larvae. Cell Stress Chaperones 2(1):60–71 10.1379/1466-1268(1997)002<0060:dcohoi>2.3.co;2 PMC312981 9250396

[39] Araújo MB, Ferri-Yáñez F, Bozinovic F, Marquet PA, Valladares F, Chown SL (2013) Heat freezes niche evolution. Ecol Lett 16(9):1206–1219 10.1111/ele.12155 23869696

[40] Pörtner HO (2002) Climate variations and the physiological basis of temperature dependent biogeography: systemic to molecular hierarchy of thermal tolerance in animals. Comp Biochem Physiol Mol Integr Physiol 132(4):739–761 10.1016/s1095-6433(02)00045-4 12095860

[41] Frederich M, Pörtner HO (2000) Oxygen limitation of thermal tolerance defined by cardiac and ventilatory performance in spider crab, *Maja squinado*. Am J Physiology-Regulatory Integr Comp Physiol 279(5):R1531–R1538 10.1152/ajpregu.2000.279.5.R1531 11049833

[42] Pörtner H-O, Zielinski S (1998) Environmental constraints and the physiology of performance in squids. S Afr J Mar Sci 20(1):207–221

[43] Brown JH, Gillooly JF, Allen AP, Savage VM, West GB (2004) Toward a Metabolic Theory of Ecology. Ecology 85(7):1771–1789

[44] Pörtner HO, Knust R (2007) Climate Change Affects Marine Fishes Through the Oxygen Limitation of Thermal Tolerance. Science 315(5808):95–97 10.1126/science.1135471 17204649

[45] Cutaneous active vasodilation as a heat loss thermoeffector (2018) Handbook of clinical neurology. Elsevier 156:193–209 10.1016/B978-0-444-63912-7.00012-6 30454590

[46] Koch F, Thom U, Albrecht E, Weikard R, Nolte W et al (2019) Heat stress directly impairs gut integrity and recruits distinct immune cell populations into the bovine intestine. Proc of the Natl Acad of Sci. 116(21):10333–38 10.1073/pnas.1820130116 PMC6535017 31064871

[47] Hall DM, Buettner GR, Oberley LW, Xu L, Matthes RD, Gisolfi CV (2001) Mechanisms of circulatory and intestinal barrier dysfunction during whole body hyperthermia. Am J Physiol Heart Circ Physiol 280(2):H509–H521 10.1152/ajpheart.2001.280.2.H509 11158946

[48] Somero GN (2004) Adaptation of enzymes to temperature: searching for basic strategies. Comp Biochem Physiol B Biochem Mol Biol 139(3):321–333 10.1016/j.cbpc.2004.05.003 15544958

[49] Dong Y, Liao M, Meng X, Somero GN (2018) Structural flexibility and protein adaptation to temperature: Molecular dynamics analysis of malate dehydrogenases of marine molluscs. Proc Natl Acad Sci U S A 115(6):1274–1279 10.1073/pnas.1718910115 PMC5819447 29358381

[50] Arnoldi J-F, Jackson AL, Peralta-Maraver I, Payne NL (2025) A universal thermal performance curve arises in biology and ecology. Proc Natl Acad Sci U S A 122(43):e2513099122 10.1073/pnas.2513099122 PMC12582339 41123997

[51] Sinclair BJ, Marshall KE, Sewell MA, Levesque DL, Willett CS et al (2016) Can we predict ectotherm responses to climate change using thermal performance curves and body temperatures? Ecol Lett 19(11):1372–1385 10.1111/ele.12686 27667778

[52] Arroyo JI, Díez B, Kempes CP, West GB, Marquet PA (2022) A general theory for temperature dependence in biology. Proc Natl Acad Sci U S A 119(30):e2119872119 10.1073/pnas.2119872119 PMC9335213 35858416

[53] Tewksbury JJ, Huey RB, Deutsch CA (2008) Putting the Heat on Tropical Animals. Science 320(5881):1296–1297 10.1126/science.1159328 18535231

[54] Buckley LB, Hurlbert AH, Jetz W (2012) Broad-scale ecological implications of ectothermy and endothermy in changing environments. Glob Ecol Biogeogr 21(9):873–885

[55] Chen I-C, Hill JK, Ohlemüller R, Roy DB, Thomas CD (2011) Rapid Range Shifts of Species Associated with High Levels of Climate Warming. Science 333(6045):1024–1026 10.1126/science.1206432 21852500

[56] Parmesan C, Ryrholm N, Stefanescu C, Hill JK, Thomas CD et al (1999) Poleward shifts in geographical ranges of butterfly species associated with regional warming. Nature 399(6736):579–583

[57] Moritz C, Patton JL, Conroy CJ, Parra JL, White GC, Beissinger SR (2008) Impact of a Century of Climate Change on Small-Mammal Communities in Yosemite National Park, USA. Science 322(5899):261–264 10.1126/science.1163428 18845755

[58] Lenoir J, Bertrand R, Comte L, Bourgeaud L, Hattab T et al (2020) Species better track climate warming in the oceans than on land. Nat Ecol Evol 4(8):1044–1059 10.1038/s41559-020-1198-2 32451428

[59] Sandmeier FC (2024) Quantification of thermal acclimation in immune functions in ectothermic animals. Biol 13(3) 10.3390/biology13030179 PMC10968454 38534449

[60] Demas GE, Zysling DA, Beechler BR, Muehlenbein MP, French SS (2011) Beyond phytohaemagglutinin: assessing vertebrate immune function across ecological contexts. J Anim Ecol 80(4):710–730 10.1111/j.1365-2656.2011.01813.x 21401591

[61] Baker SJ, Merchant ME (2018) Antibacterial properties of plasma from the prairie rattlesnake (*Crotalus viridis*). Dev Comp Immunol 84:273–278 10.1016/j.dci.2018.03.002 29510163

[62] Bakewell L, Kelehear C, Graham S p (2021) Impacts of temperature on immune performance in a desert anuran (Anaxyrus punctatus). J Zool 315(1):49–57

[63] Moretti EH, Titon SCM, Titon B, Marques FS, Gomes FR (2019) Thermal sensitivity of innate immune response in three species of *Rhinella* toads. Comp Biochem Physiol Mol Integr Physiol 237:110542 10.1016/j.cbpa.2019.110542 31419577

[64] Ferguson LV, Heinrichs DE, Sinclair BJ (2016) Paradoxical acclimation responses in the thermal performance of insect immunity. Oecologia 181(1):77–85 10.1007/s00442-015-3529-6 26846428

[65] Xu D-L, Wang Y (2023) Effect of gradual increase and decrease in temperature on innate, cellular and humoral immunity in striped hamsters. Mamm Biol 103(3):265–276

[66] Xu D-L, Xu M-M, Wang D-H (2019) Effect of temperature on antioxidant defense and innate immunity in Brandt’s voles. Zool Res 40(4):305–316 10.24272/j.issn.2095-8137.2019.045 PMC6680122 31310064

[67] Lucore JM, Beehner JC, White AF, Sinclair LF, Martins VA et al (2024) High temperatures are associated with decreased immune system performance in a wild primate. Sci Adv 10(48):eadq6629 10.1126/sciadv.adq6629 PMC11619714 39612329

[68] Leijh PCJ, Van Den MTh B, Van Furth R (1981) Kinetics of Phagocytosis and Intracellular Killing of Staphytococcus aureus and Escherichia coli by Human Monocytes. Scand J Immunol 13(2):159–174 10.1111/j.1365-3083.1981.tb00122.x 7015485

[69] Roberts N, Steigbigel R (1977) Hyperthermia and human leukocyte functions: effects on response of lymphocytes to mitogen and antigen and bactericidal capacity of monocytes and neutrophils. Infect Immun 18(3) 10.1128/iai.18.3.673-679.1977 PMC421288 412788

[70] Peterson PK, Verhoef J, Sabath LD, Quie PG (1976) Extracellular and bacterial factors influencing staphylococcal phagocytosis and killing by human polymorphonuclear leukocytes. Infect Immun 14(2):496–501 10.1128/iai.14.2.496-501.1976 PMC420912 971959

[71] Sebag J, Reed WP, Williams RC J (1977) Effect of temperature on bacterial killing by serum and by polymorphonuclear leukocytes. Infect Immun 16(3):947–954 10.1128/iai.16.3.947-954.1977 PMC421056 19356

[72] Hatzfeld-Charbonnier AS, Lasek A, Castera L, Gosset P, Velu T et al (2007) Influence of heat stress on human monocyte-derived dendritic cell functions with immunotherapeutic potential for antitumor vaccines. J Leukoc Biol 81(5):1179–1187 10.1189/jlb.0506347 PMC2254491 17311933

[73] Jimenez C, Melin B, Savourey G, Launay J-C, Alonso A, Mathieu J (2007) Effects of passive hyperthermia versus exercise-induced hyperthermia on immune responses: hormonal implications. Eur Cytokine Netw 18(3):154–161 10.1684/ecn.2007.0101 17823084

[74] Pilch W, Szarek M, Olga C-L, Anna P, Żychowska M et al (2023) The effects of a single and a series of Finnish sauna sessions on the immune response and HSP-70 levels in trained and untrained men. Int J Hyperth 40(1):2179672 10.1080/02656736.2023.2179672 36813265

[75] Webster JI, Tonelli L, Sternberg EM (2002) Neuroendocrine Regulation of Immunity. Annu Rev Immunol 20(1):125–163 10.1146/annurev.immunol.20.082401.104914 11861600

[76] Matzinger P (2002) The Danger Model: A Renewed Sense of Self. Science 296(5566):301–305 10.1126/science.1071059 11951032

[77] Chen R, Zou J, Liu J, Kang R, Tang D (2025) DAMPs in the immunogenicity of cell death. Mol Cell 85(20):3874–3889 10.1016/j.molcel.2025.09.007 41106375

[78] Ma M, Jiang W, Zhou R (2024) DAMPs and DAMP-sensing receptors in inflammation and diseases. Immunity 57(4):752–771 10.1016/j.immuni.2024.03.002 38599169

[79] Zininga T, Ramatsui L, Shonhai A, Zininga T, Ramatsui L, Shonhai A (2018) Heat shock proteins as immunomodulants. Mol 23(11) 10.3390/molecules23112846 PMC6278532 30388847

[80] Asea A, Rehli M, Kabingu E, Boch JA, Baré O et al (2002) Novel Signal Transduction Pathway Utilized by Extracellular HSP70. J Biol Chem 277(17):15028–15034 10.1074/jbc.M200497200 11836257

[81] Quintana FJ, Cohen IR (2011) The HSP60 immune system network. Trends Immunol 32(2):89–95 10.1016/j.it.2010.11.001 21145789

[82] Andrés CMC, de la Pérez JM, Juan CA, Plou FJ, Pérez-Lebeña E (2022) The Role of Reactive Species on Innate Immunity. Vaccines 10(10):1735 10.3390/vaccines10101735 PMC9609844 36298601

[83] Finkel T, Holbrook NJ (2000) Oxidants, oxidative stress and the biology of ageing. Nature 408(6809):239–247 10.1038/35041687 11089981

[84] Zanini G, Selleri V, Lopez Domenech S, Malerba M, Nasi M et al (2023) Mitochondrial DNA as inflammatory DAMP: a warning of an aging immune system? Biochem Soc Trans 51(2):735–745 10.1042/BST20221010 37013978

[85] Green DR, Kroemer G (2004) The Pathophysiology of Mitochondrial Cell Death. Science 305(5684):626–629 10.1126/science.1099320 15286356

[86] West AP, Shadel GS (2017) Mitochondrial DNA in innate immune responses and inflammatory pathology. Nat Rev Immunol 17(6):363–375 10.1038/nri.2017.21 PMC7289178 28393922

[87] Gehrke N, Mertens C, Zillinger T, Wenzel J, Bald T et al (2013) Oxidative Damage of DNA Confers Resistance to Cytosolic Nuclease TREX1 Degradation and Potentiates STING-Dependent Immune Sensing. Immunity 39(3):482–495 10.1016/j.immuni.2013.08.004 23993650

[88] Kirkinezos IG, Moraes CT (2001) Reactive oxygen species and mitochondrial diseases. Semin Cell Dev Biol 12(6):449–457 10.1006/scdb.2001.0282 11735379

[89] Ohlsson L, Hall A, Lindahl H, Danielsson R, Gustafsson A et al (2020) Increased level of circulating cell-free mitochondrial DNA due to a single bout of strenuous physical exercise. Eur J Appl Physiol 120(4):897–905 10.1007/s00421-020-04330-8 PMC7125245 32088743

[90] Trumpff C, Michelson J, Lagranha CJ, Taleon V, Karan KR et al (2021) Stress and circulating cell-free mitochondrial DNA: A systematic review of human studies, physiological considerations, and technical recommendations. Mitochondrion 59:225–245 10.1016/j.mito.2021.04.002 PMC8418815 33839318

[91] Horn P, Kalz A, Lim CL, Pyne D, Saunders P et al (2007) Exercise-recruited NK cells display exercise-associated eHSP-70. Exerc Immunol Rev 13:100–111 18198664

[92] Gibson OR, Dennis A, Parfitt T, Taylor L, Watt PW, Maxwell NS (2014) Extracellular Hsp72 concentration relates to a minimum endogenous criteria during acute exercise-heat exposure. Cell Stress Chaperones 19(3):389–400 10.1007/s12192-013-0468-1 PMC3982022 24085588

[93] Sapolsky RM, Romero LM, Munck AU (2000) How Do Glucocorticoids Influence Stress Responses? Integrating Permissive, Suppressive, Stimulatory, and Preparative Actions*. Endocr Rev 21(1):55–89 10.1210/edrv.21.1.0389 10696570

[94] Dhabhar FS (2009) Enhancing versus Suppressive Effects of Stress on Immune Function: Implications for Immunoprotection and Immunopathology. Neuroimmunomodulation 16(5):300–317 10.1159/000216188 PMC2790771 19571591

[95] Glaser R, Kiecolt-Glaser JK (2005) Stress-induced immune dysfunction: implications for health. Nat Rev Immunol 5(3):243–251 10.1038/nri1571 15738954

[96] Padgett DA, Glaser R (2003) How stress influences the immune response. Trends Immunol 24(8):444–448 10.1016/s1471-4906(03)00173-x 12909458

[97] Cain DW, Cidlowski JA (2017) Immune regulation by glucocorticoids. Nat Rev Immunol 17(4):233–247 10.1038/nri.2017.1 PMC9761406 28192415

[98] Elenkov IJ, Chrousos GP (2002) Stress Hormones, Proinflammatory and Antiinflammatory Cytokines, and Autoimmunity. Ann N Y Acad Sci 966(1):290–303 10.1111/j.1749-6632.2002.tb04229.x 12114286

[99] Elenkov IJ (2007) Effects of Catecholamines on the Immune Response. NeuroImmune Biol 7:189–206

[100] Sheridan JF, Dobbs C, Jung J, Chu X, Konstantinos A et al (1998) Stress-induced neuroendocrine modulation of viral pathogenesis and immunity. Ann N Y Acad Sci 840:803–808 10.1111/j.1749-6632.1998.tb09618.x PMC1351103 9629306

[101] Stone AA, Bovbjerg DH, Neale JM, Napoli A, Valdimarsdottir H et al (1992) Development of Common Cold Symptoms Following Experimental Rhinovirus Infection is Related to Prior Stressful Life Events. Behav Med 18(3):115–120 10.1080/08964289.1992.9936961 1330102

[102] Follenius M, Brandenberger G, Oyono S, Candas V (1982) Cortisol as a sensitive index of heat-intolerance. Physiology Behavior 29(3):509–513 10.1016/0031-9384(82)90274-8 7178257

[103] Hu Y, Jin H, Du X, Xiao C, Luo D et al (2007) Effects of Chronic Heat Stress on Immune Responses of the Foot-and-Mouth Disease DNA Vaccination. DNA Cell Biol 26(8):619–626 10.1089/dna.2007.0581 17688414

[104] Meng D, Hu Y, Xiao C, Wei T, Zou Q, Wang M (2013) Chronic Heat Stress Inhibits Immune Responses to H5N1 Vaccination through Regulating CD4 + CD25+Foxp3 + Tregs. BioMed Res Int 2013(1):160859 10.1155/2013/160859 PMC3787559 24151582

[105] Bagath M, Krishnan G, Devaraj C, Rashamol VP, Pragna P et al (2019) The impact of heat stress on the immune system in dairy cattle: A review. Res Vet Sci 126:94–102 10.1016/j.rvsc.2019.08.011 31445399

[106] Safa S, Kargar S, Moghaddam GA, Ciliberti MG, Caroprese M (2019) Heat stress abatement during the postpartum period: effects on whole lactation milk yield, indicators of metabolic status, inflammatory cytokines, and biomarkers of the oxidative stress. J Anim Sci 97(1):122–132 10.1093/jas/sky408 PMC6313133 30346551

[107] Wojtas K, Cwynar P, Kołacz R (2014) Effect of thermal stress on physiological and blood parameters in merino sheep. Bull Vet Inst Pulawy 58(2):283–288

[108] Yadav B, Mohammed AN, Graham B, Bhattacharya A, Yadav JS (2024) Chronic Heat Exposure Modulates Innate and Adaptive Immune Responses in Firefighters. Environments 11(6):131

[109] Grissom N, Bhatnagar S (2009) Habituation to repeated stress: Get used to it. Neurobiol Learn Mem 92(2):215–224 10.1016/j.nlm.2008.07.001 PMC2773683 18667167

[110] Ju X-H, Xu H-J, Yong Y-H, An L-L, Jiao P-R, Liao M (2014) Heat stress upregulation of Toll-like receptors 2/4 and acute inflammatory cytokines in peripheral blood mononuclear cell (PBMC) of Bama miniature pigs: an *in vivo* and *in vitro* study. Animal 8(9):1462–1468 10.1017/S1751731114001268 24912383

[111] Roh JS, Sohn DH (2018) Damage-Associated Molecular Patterns in Inflammatory Diseases. Immune Netw 18(4):e27 10.4110/in.2018.18.e27 PMC6117512 30181915

[112] Huang Q-Q, Sobkoviak R, Jockheck-Clark AR, Shi B, Mandelin AM et al (2009) Heat shock protein 96 is elevated in rheumatoid arthritis and activates macrophages primarily via TLR2 signaling. J Immunol 182(8):4965–4973 10.4049/jimmunol.0801563 PMC2814438 19342676

[113] Martin CA, Carsons SE, Kowalewski R, Bernstein D, Valentino M, Santiago-Schwarz F (2003) Aberrant extracellular and dendritic cell (DC) surface expression of heat shock protein (hsp)70 in the rheumatoid joint: possible mechanisms of hsp/DC-mediated cross-priming. J Immunol 171(11):5736–5742 10.4049/jimmunol.171.11.5736 14634081

[114] Caielli S, Athale S, Domic B, Murat E, Chandra M et al (2016) Oxidized mitochondrial nucleoids released by neutrophils drive type I interferon production in human lupus. J Exp Med 213(5):697–713 10.1084/jem.20151876 PMC4854735 27091841

[115] Franceschi C, Bonafè M, Valensin S, Olivieri F, De Luca M et al (2000) Inflamm-aging: An Evolutionary Perspective on Immunosenescence. Ann N Y Acad Sci 908(1):244–254 10.1111/j.1749-6632.2000.tb06651.x 10911963

[116] Fulop T, Witkowski JM, Olivieri F, Larbi A (2018) The integration of inflammaging in age-related diseases. Semin Immunol 40:17–35 10.1016/j.smim.2018.09.003 30287177

[117] López-Otín C, Blasco MA, Partridge L, Serrano M, Kroemer G (2023) Hallmarks of aging: An expanding universe. Cell 186(2):243–278 10.1016/j.cell.2022.11.001 36599349

[118] Pinti M, Cevenini E, Nasi M, De Biasi S, Salvioli S et al (2014) Circulating mitochondrial DNA increases with age and is a familiar trait: Implications for inflamm-aging. Eur J Immunol 44(5):1552–1562 10.1002/eji.201343921 24470107

[119] Chen S, Liu Y, Yi Y, Zheng Y, Yang J et al (2025) Long-term impacts of heatwaves on accelerated ageing. Nat Clim Chang 1–8

[120] Zeng MY, Inohara N, Nuñez G (2017) Mechanisms of inflammation-driven bacterial dysbiosis in the gut. Mucosal Immunol 10(1):18–26 10.1038/mi.2016.75 PMC5788567 27554295

[121] Hughes ER, Winter MG, Duerkop BA, Spiga L, de Carvalho TF et al (2017) Microbial respiration and formate oxidation as metabolic signatures of inflammation-associated dysbiosis. Cell Host Microbe 21(2):208–219 10.1016/j.chom.2017.01.005 PMC5313043 28182951

[122] Sepulveda J, Moeller AH (2020) The Effects of Temperature on Animal Gut Microbiomes. Front Microbiol 11:384 10.3389/fmicb.2020.00384 PMC7076155 32210948

[123] Corrêa-Oliveira R, Fachi JL, Vieira A, Sato FT, Vinolo MAR (2016) Regulation of immune cell function by short-chain fatty acids. Clinical Translational Immunology 5(4):e73 10.1038/cti.2016.17 PMC4855267 27195116

[124] Flint HJ, Scott,Karen P, Duncan, Sylvia H, Louis, Petra, and, Forano E (2012) Microbial degradation of complex carbohydrates in the gut. Gut Microbes 3(4):289–306 10.4161/gmic.19897 PMC3463488 22572875

[125] Rizzatti G, Lopetuso LR, Gibiino G, Binda C, Gasbarrini A (2017) Proteobacteria: A Common Factor in Human Diseases. BioMed Res Int 2017(1):9351507 10.1155/2017/9351507 PMC5688358 29230419

[126] Gray JS, Dautel H, Estrada-Peña A, Kahl O, Lindgren E (2009) Effects of Climate Change on Ticks and Tick-Borne Diseases in Europe. Interdiscip Perspect Infect Dis 2009(1):593232 10.1155/2009/593232 PMC2648658 19277106

[127] El-Sayed A, Kamel M (2020) Climatic changes and their role in emergence and re-emergence of diseases. Res J Environ Sci 27(18):22336–22352 10.1007/s11356-020-08896-w PMC7187803 32347486

[128] Harvell CD, Mitchell CE, Ward JR, Altizer S, Dobson AP et al (2002) Climate Warming and Disease Risks for Terrestrial and Marine Biota. Science 296(5576):2158–2162 10.1126/science.1063699 12077394

[129] Mora C, McKenzie T, Gaw IM, Dean JM, von Hammerstein H et al (2022) Over half of known human pathogenic diseases can be aggravated by climate change. Nat Clim Chang 12(9):869–875 10.1038/s41558-022-01426-1 PMC9362357 35968032

[130] Simon AK, Hollander GA, McMichael A (2015) Evolution of the immune system in humans from infancy to old age. Proc. R. Soc. B. 282(1821):20143085 10.1098/rspb.2014.3085 PMC4707740 26702035

[131] Swaminathan A, Lucas RM, Harley D, McMichael AJ (2014) Will Global Climate Change Alter Fundamental Human Immune Reactivity: Implications for Child Health? Children 1(3):403–423 10.3390/children1030403 PMC4928742 27417487

[132] Padgett DA, Sheridan JF, Dorne J, Berntson GG, Candelora J, Glaser R (1998) Social stress and the reactivation of latent herpes simplex virus type 1. Proc. Natl. Acad. Sci.* U. S. A.* 95(12):7231–35 10.1073/pnas.95.12.7231 PMC22787 9618568

[133] Wonnacott KM, Bonneau RH (2002) The Effects of Stress on Memory Cytotoxic T Lymphocyte-Mediated Protection against Herpes Simplex Virus Infection at Mucosal Sites. Brain Behav Immun 16(2):104–117 10.1006/brbi.2001.0624 11908921

[134] Cohen F, Kemeny ME, Kearney KA, Zegans LS, Neuhaus JM, Conant MA (1999) Persistent Stress as a Predictor of Genital Herpes Recurrence. Arch Intern Med 159(20):2430–2436 10.1001/archinte.159.20.2430 10665891

[135] Padgett DA, Marucha PT, Sheridan JF (1998) Restraint Stress Slows Cutaneous Wound Healing in Mice. Brain Behav Immun 12(1):64–73 10.1006/brbi.1997.0512 9570862

[136] Kiecolt-Glaser JK, Marucha PT, Mercado AM, Malarkey WB, Glaser R (1995) Slowing of wound healing by psychological stress. Lancet 346(8984):1194–1196 10.1016/s0140-6736(95)92899-5 7475659

[137] dos Santos-Silva MA, Trajano ETL, Schanuel FS, Monte-Alto-Costa A (2017) Heat delays skin wound healing in mice. Exp Biol Med 242(3):258–266 10.1177/1535370216675066 PMC5384502 27798118

[138] Coates SJ, Enbiale W, Davis MDP, Andersen LK (2020) The effects of climate change on human health in Africa, a dermatologic perspective: a report from the International Society of Dermatology Climate Change Committee. Int J Dermatol 59(3):265–278 10.1111/ijd.14759 31970754

[139] Zipple MN, Vogt CC, Sheehan MJ (2023) Re-wilding model organisms: Opportunities to test causal mechanisms in social determinants of health and aging. Neurosci Biobehav Rev 152:105238 10.1016/j.neubiorev.2023.105238 PMC10527394 37225063

[140] Kearney MR, Matzelle A, Helmuth B (2012) Biomechanics meets the ecological niche: the importance of temporal data resolution. J Exp Biol 215(6):922–933 10.1242/jeb.059634 22357586

[141] Campos FA, Fedigan LM (2009) Behavioral adaptations to heat stress and water scarcity in white-faced capuchins (Cebus capucinus) in Santa Rosa National Park, Costa Rica. Am J Phys Anthropol 138(1):101–111 10.1002/ajpa.20908 18711741

[142] Iii JWC, Krausman PR, Rosenstock SS, Turner JC (2006) Mechanisms of Thermoregulation and Water Balance in Desert Ungulates. Wildl Soc Bull 34(3):570–581

[143] Alberts SC, Altmann J (2012) The Amboseli Baboon Research Project: 40 Years of Continuity and Change. In: Kappeler PM (ed) Long-Term Field Studies of Primates. Berlin, Heidelberg: Springer, DP Watts, pp 261–287

[144] Lonsdorf EV, Travis DA, Raphael J, Kamenya S, Lipende I et al (2022) The Gombe Ecosystem Health Project: 16 Years of Program Evolution and Lessons Learned. Am J Primatol 84:4–5 10.1002/ajp.23300 PMC8727649 34223656

[145] Lucore JM, Marshall AJ, Brosnan SF, Benítez ME (2022) Validating urinary neopterin as a biomarker of immune response in captive and wild capuchin monkeys. Front Vet Sci 9(918036) 10.3389/fvets.2022.918036 PMC9326447 35909690

[146] Higham JP, Stahl-Hennig C, Heistermann M (2020) Urinary suPAR: a non-invasive biomarker of infection and tissue inflammation for use in studies of large free-ranging mammals. R Soc Open Sci 7(2):191825 10.1098/rsos.191825 PMC7062102 32257339

